# Geographic accessibility to primary healthcare centers in Mozambique

**DOI:** 10.1186/s12939-016-0455-0

**Published:** 2016-10-18

**Authors:** António dos Anjos Luis, Pedro Cabral

**Affiliations:** 1 Universidade Católica de Moçambique, Beira, Moçambique; 2 NOVA IMS, Universidade Nova de Lisboa, 1070-312 Lisboa, Portugal

**Keywords:** Accessibility, Health centers, Service area, Mozambique, Geographic information systems

## Abstract

**Background:**

Access to healthcare services has an essential role in promoting health equity and quality of life. Knowing where the places are and how much of the population is covered by the existing healthcare network is important information that can be extracted from Geographical Information Systems (GIS) and used in effective healthcare planning. The aim of this study is to measure the geographic accessibility of population to existing Healthcare Centers (HC), and to estimate the number of persons served by the health network of Mozambique.

**Methods:**

Health facilities’ locations together with population, elevation, and ancillary data were used to model accessibility to HC using GIS. Two travel time scenarios used by population to attend HC were considered: (1) Driving and; and (2) Walking. Estimates of the number of villages and people located in the region served, i.e. within 60 min from an HC, and underserved area, i.e. outside 60 min from an HC, are provided at national and province level.

**Results:**

The findings from this study highlight accessibility problems, especially in the walking scenario, in which 90.2 % of Mozambique was considered an underserved area. In this scenario, Maputo City (69.8 %) is the province with the greatest coverage of HC. On the other hand, Tete (93.4 %), Cabo Delgado (93 %) and Gaza (92.8 %) are the provinces with the most underserved areas. The driving scenario was less problematic, with about 66.9 % of Mozambique being considered a served area. We also found considerable regional disparities at the province level for this scenario, ranging from 100 % coverage in Maputo City to 48.3 % in Cabo Delgado. In terms of population coverage we found that the problem of accessibility is more acute in the walking scenario, in which about 67.3 % of the Mozambican population is located in underserved areas. For the driving scenario, only 6 % of population is located in underserved areas.

**Conclusions:**

This study highlights critical areas in Mozambique in which HC are lacking when assessed by walking and driving travel time distance. The majority of Mozambicans are located in underserved areas in the walking scenario. The mapped outputs may have policy implications and can be used for future decision making processes and analysis.

**Trial registration:**

Not applicable.

## Background

Universal health coverage has been considered a pillar of sustainable development and global security [1]. Thus, health related facilities should be universally available, accessible, acceptable, appropriate, and of good quality (AAAQ framework) [2]. In public health there is a direct link between the distance patients travel to access health and the reduction of ill health and suffering in a country [3]. Patients tend to use health facilities more if they are located close to them than if they are far way [4]. The issue of distance of the patients to the centers is seen as one of the main determinants of use of health services [5]. In third world countries the distance covered by patients is usually greater than in developed world countries, in which healthcare facilities are more accessible. This has an important impact on the quality of life of these countries [5]. Accessibility to healthcare is the capability of a population to obtain a specified set of healthcare services [6]. Reflecting the equilibrium between characteristics and expectations of the providers and the clients, quality care has been conceptualized in four dimensions of access [7]: (1) geographic accessibility– the physical distance or travel time to the potential user; (2) availability – having the adequate type of care for who is needing it; (3) financial accessibility – willingness and ability of users to pay for services; (4) acceptability – response of the health services providers to the social and cultural individual expectations and communities in general. Identifying different levels of spatial accessibility to healthcare services in a certain area allows decision makers to understand the impacts of opening, closing, changing location or modifying the services offered by existing facilities [8].

Currently, several advanced methodological approaches are used to estimate health accessibility, such as gravity, kernel density, and catchment area models [9]. However, the conventional and most common techniques used to calculate accessibility in public health research are still the Euclidean and network distance [4]. Euclidean distance techniques describe a location’s relationship to a source or a set of sources based on the straight-line distance [10]. Networked distance is the physical travel path or road to reach the destination [11]. The constraint of the Euclidian distance is that it does not take into account physical barriers to movements and transportation routes, thereby underestimating the real travel distance [12, 13]. Because of the sparse road network and natural obstacles, such as water and mountains, it is not adequate to estimate accessibility using Euclidian distances [14]. On the contrary, when road networks are used, the accessibility tends to be greater in places where there are many good road networks in combination with the presence of health facilities [15].

The World Health Organization (WHO) suggests the use of travel time, instead of distance, to assess healthcare services because this method takes into consideration the conditions of the roads and the means of transport [16]. There is no universally accepted range of time for allowing people to travel for medical care. Some authors consider the range of 30 min for access to patient care as reduced [17]. Others state that people living at more than 45 min from healthcare facilities are more likely to be marginalized; and there is a group of authors that consider one hour as an adequate (which agrees with the opinion of ambulance drivers [18]).

The use of GIS in public health has had a tremendous growth as result of the availability of various information technology services and software, and is currently being considered useful to the understanding and treatment of health problems in different geographic areas [19]. A considerable number of studies concerned with measures of access to healthcare services were developed as a result of the availability of GIS in health organizations and the increasing availability of spatial disaggregate data [20].

**Fig. 1:**
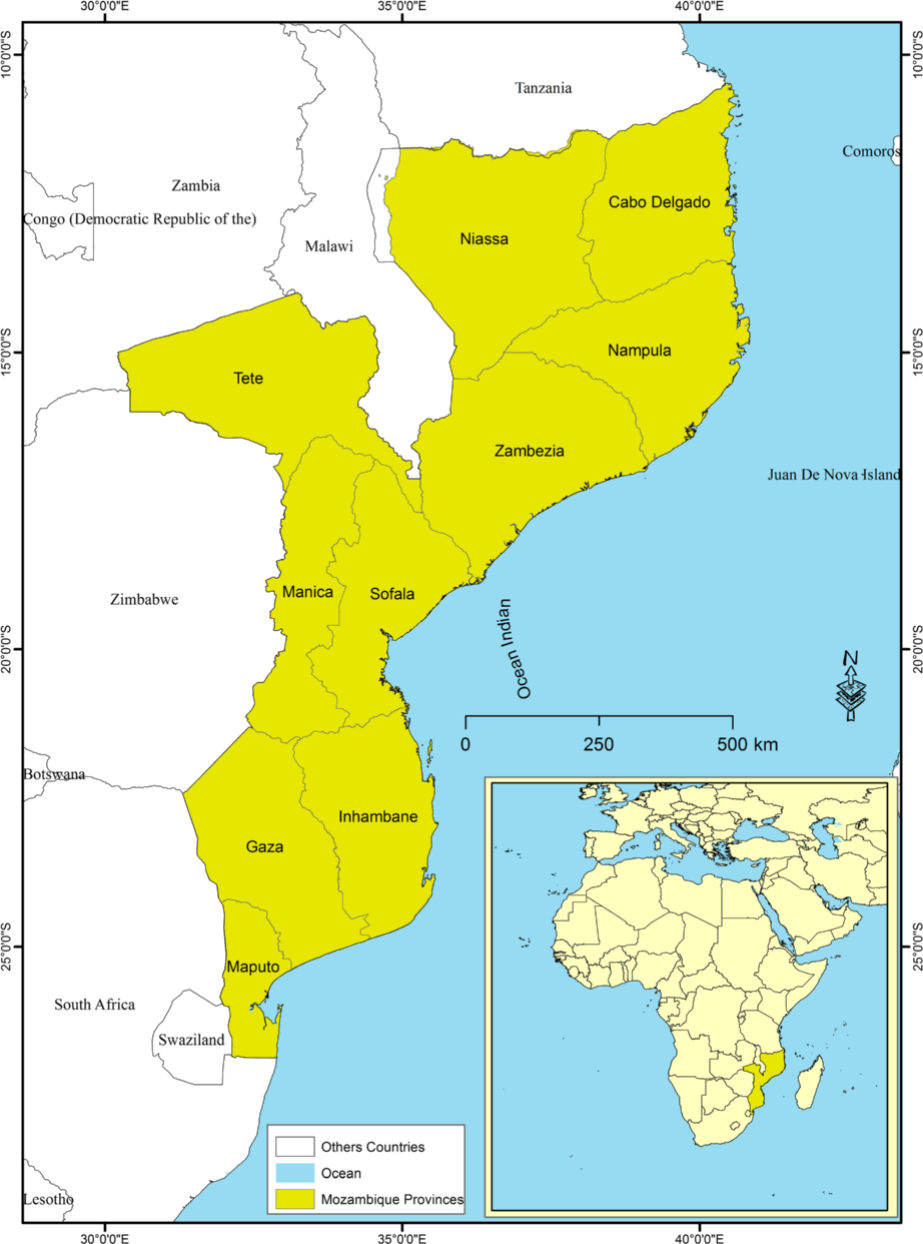
Mozambique’s Location

Mozambique is located in the Southern Region of Africa, and has borders with Tanzania (North), Malawi, Zambia and Zimbabwe (West), and South Africa and Swaziland (South). The country has an area of 799,380 km^2^, with a long eastern shoreline on the Indian Ocean (Fig. 1). The total estimated population for 2012 is 23.4 million, spread over 11 provinces, including Maputo City, which has provincial status [21]. Mozambique ranks 180th position out of 188 countries in the Human Development Index 2015, being classified as a low development country [22]. Over 70% of the population lives in rural areas and below the poverty line. Although agriculture is the main source of household food and income, the production at the household level is often insufficient to maintain food security [23]. The country’s high poverty levels, the chronic malnutrition in a context of marked food insecurity, the low levels of education of women, the poor access to clean water and poor sanitation, and the limited access to quality health services are the main determinants of health status and burden of disease in Mozambique [24]. The epidemiological situation of Mozambique is largely pre-transitional, i.e. dominated by communicable diseases, namely malaria, HIV/AIDS, diarrhea, acute respiratory infections and tuberculosis, but with a pronounced rise of non-communicable diseases (cardiovascular diseases, injuries, cancers, etc.), particularly in urban areas [21].

Strengthening health systems and ensuring increasing equitable access to health services, and building management capacity in the public health sector as well as expanding its coverage are top strategic priorities for the country [25]. The health system in Mozambique is organized in four levels, namely [26]: a) the primary level, comprising urban and rural HC; b) the secondary level, comprising general, rural, and district hospitals; c) the tertiary level, comprising the hospitals of the provincial capitals; and d) the quaternary level, represented by the central hospitals of Beira, Nampula, and Maputo and the Specialized Hospitals. The primary level of the system encompasses a set of basic actions to solve the most common problems in the community. Between 70 and 80 % of the problems that drive the demand for healthcare can be solved at this level.

The focus of this paper is the primary level of healthcare facilities. The secondary level is more differentiated and developed, supporting the primary level technical and organizational problems. This level solves more complex situations than the primary level, referring to other levels of care (tertiary and even quaternary) the solution of situations that go beyond the scope of its competence. The secondary level hospitals have as secondary function to dispense healthcare and constitutes the first level of referral for patients who cannot find solution to their health problems in health centers of their areas of influence. Provincial hospitals provide tertiary healthcare and are the reference level for patients who cannot find a solutions for their health problems in district, rural, and general hospitals, as well as for patients from HC located in the vicinity of the provincial hospital, which has neither a rural hospital nor general hospital to which they can be referred. The quaternary level has a regional and national basis, and is in charge of the three existing central hospitals in the cities of Maputo, Beira, and Nampula. Each of these central hospitals is responsible for one national territory and for the psychiatric hospitals of Infulene and Nampula.

It is hypothesized that a lack of health facilities close to people is a major obstacle to reaching health facilities and can inhibit access [27]. Long travel times and greater distances can lead patients not to repeat the visit to the healthcare facilities [28].

The issue of distance and time as barriers to healthcare services has not been well documented in Mozambique; usually, distance has been examined as a binary variable (far/close) and there are no accessibility maps showing how far or close the communities are to the health facilities. Additionally, there has been no systematic attempt to analyze the effects of the distance barriers to healthcare in Mozambique. This study seeks to fill this knowledge gap by measuring geographical accessibility to HC facilities in Mozambique. We calculate the spatial coverage of the existing primary HC facility network using two scenarios of travel time: driving and walking. We also estimate the number of people within and outside 60 min from an HC to understand the degree of accessibility of the Mozambican population to the health network.

## Methods

The focus of this study is primary HC because these units encompass a set of basic actions to solve the most common problems in the community. The location of HC was obtained using the USAID dataset survey of year 2000. This dataset was updated to year 2016 by the authors of this study through a list provided by the Minister of Health of Mozambique. The total number of HC included in the analysis is 1,061, corresponding to 81.2 % percent of all existing HC in Mozambique. The Gridded Population of the World (GPW) data from the Global Rural–urban Mapping Project (GRUMP) projected for 2015 was used to map the population of Mozambique. These data were downloaded from the Internet [29] and consist of an estimation of human population by 2.5 arc-minute grid cells. The digital elevation model (DEM) for Mozambique was obtained from the Aster GDEM [30] with 30 m of spatial resolution. A total of 101 tiles were mosaicked in order to obtain a single DEM file for the whole country. The elevation data were used to calculate walking time with QGIS free open source software [31]. For the study area delimitation we used an administrative map produced by the National Cartography and Tele-detection Centre from Mozambique [32]. This dataset represents the administrative division of the country in three levels: provincial, district and administrative post. The road network was also obtained from the same source and was classified in three categories: main road, secondary road, and tertiary road (mostly unpaved). The mapping of road network and modeling of spatial data can be used to identify restrictions on vehicle movement [33]. After correcting the topological road network problems, this dataset was superposed with the health facilities. During this process we verified that some health facilities were too far from the road network, which could confound the analysis. To minimize this problem we updated the road network by digitizing some road segments from Google Earth [34]. These were then exported to ArcGIS software [35]. The villages and communities dataset was obtained from USAID project data of year 2000.

The accessibility analysis was carried out using the Service Area (SA) tool of Network Analyst extension from ArcGIS [35]. Two scenarios of travel time for Mozambique were created: travel time by roads and by walking. The SA was based on the driving distance by road and walking distance criteria described in Table 1. The straight-line Euclidean distance to create a buffer around the HC was initially considered as a solution to create the SA. However, this approach was not realistic from a walkability standpoint because it fails to take into account physical barriers, such as water bodies, railway lines, buildings, and other obstructions [36]. The function used to calculate driving and walking time in minutes through the road network was:

**Table 1:** Walking and driving travel times on different road types in Mozambique

Road Type	Travel Time	
	Walking	Vehicle
Primary	5 km/h (12 min/km)	80 km/h (0.75 min/km)
Secondary	4 km/h (15 min/km)	50 km/h (1.2 min/km)
Tertiary	4 km/h (15 min/km)	20 km/h (3.0 min/km)

$$ \mathrm{Length}\ \mathrm{o}\mathrm{f}\ \mathrm{the}\ \mathrm{Roads}/\mathrm{Maximum}\ \mathrm{Speed}\ \left(\mathrm{f}\mathrm{o}\mathrm{r}\ \mathrm{each}\ \mathrm{type}\ \mathrm{o}\mathrm{f}\ \mathrm{the}\ \mathrm{r}\mathrm{o}\mathrm{ad}\right)*60 $$

For determining the geographical accessibility to HC, two scenarios for travelling to the health facilities were considered (Table 1): driving time and walking time. The estimates for walking time were obtained with QGIS python plugin which uses Tobler’s hiking formula to determine the travel time along a line depending on the slope [37]. The input data were the vector layer with lines (road network) and the DEM. The fields with estimated time in minutes in forward and reverse directions were created with the default value of speed of 5 km/h. As a result of the lack of infrastructures and motorized transport services the predominant way of transport in rural Africa areas is walking [16]. Research in less developed countries, often uses walking time or travel time by public transportation to measure distance to the nearest hospital [18].

The maximum travelling time to be considered a served area was set to 60 min. Areas more than 60 min away from HC were considered underserved for both scenarios. The population should have access to a health facility within one hour of walking [16]. More than that, people will pay a high cost (financially and emotionally) to visit a healthcare center [18]. The number of villages and population were superposed with the category’s distance in order to know the villages and population served for each section of time. The number of population for each province was estimated for the two scenarios for the served and underserved areas.

## Results

**Fig. 2:**
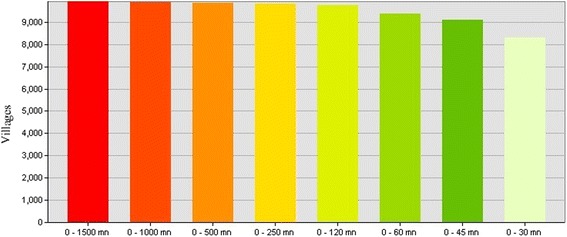
Number of villages per driving time category

**Fig. 3:**
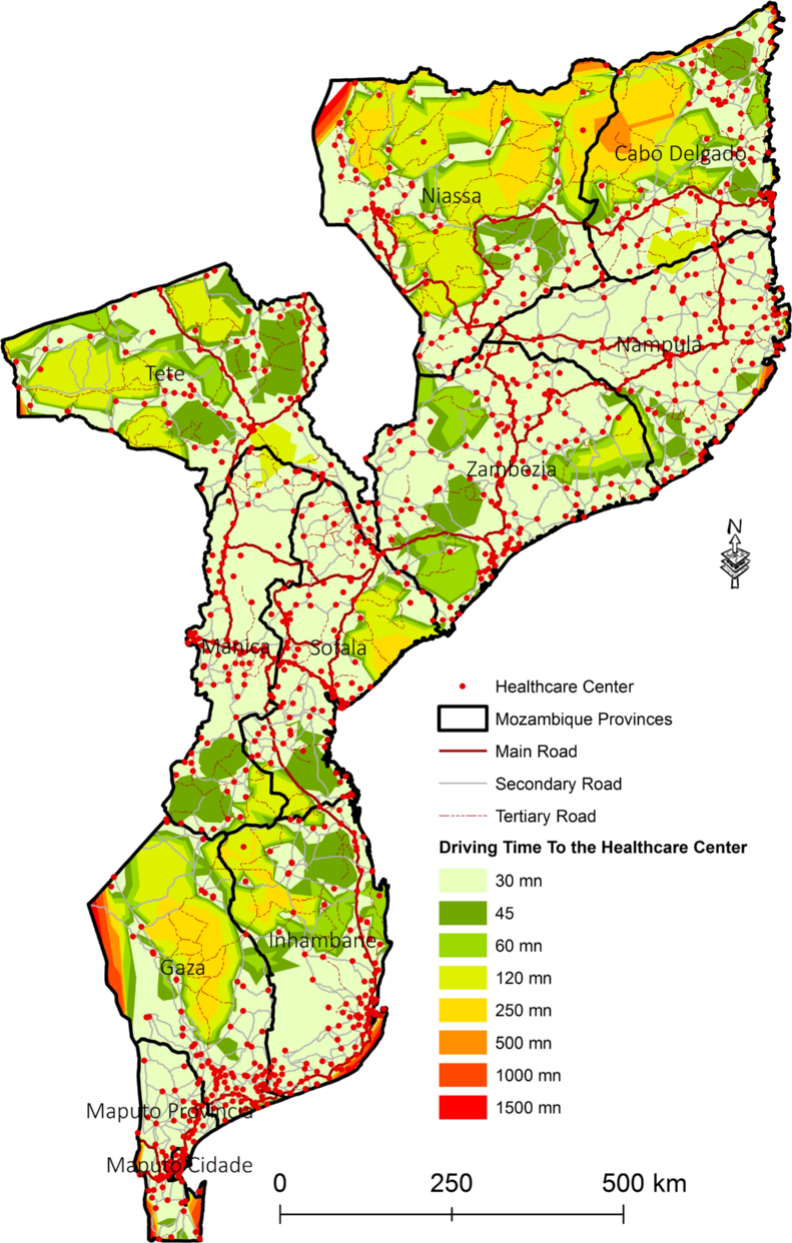
Driving time to Healthcare Centers in different time categories

For the driving scenario, the calculated catchment areas of each HC were divided in to eight categories: 30, 45, 60, 120, 250, 500, 1000, and 1500 min. The number and location of the villages served by each catchment area were obtained (Figs. 2 and 3).

The map in Fig. 3 shows that the best areas served by the health network are located mainly in the provinces of Nampula, part of the province of Zambezia, Tete, central and Northern provinces of Manica and Sofala as well as the south of Gaza, and most of the Maputo Province. In contrast, the driving travel time to HC is lowest in the provinces of Niassa, Cabo Delgado, and part of Gaza province.

**Fig. 4:**
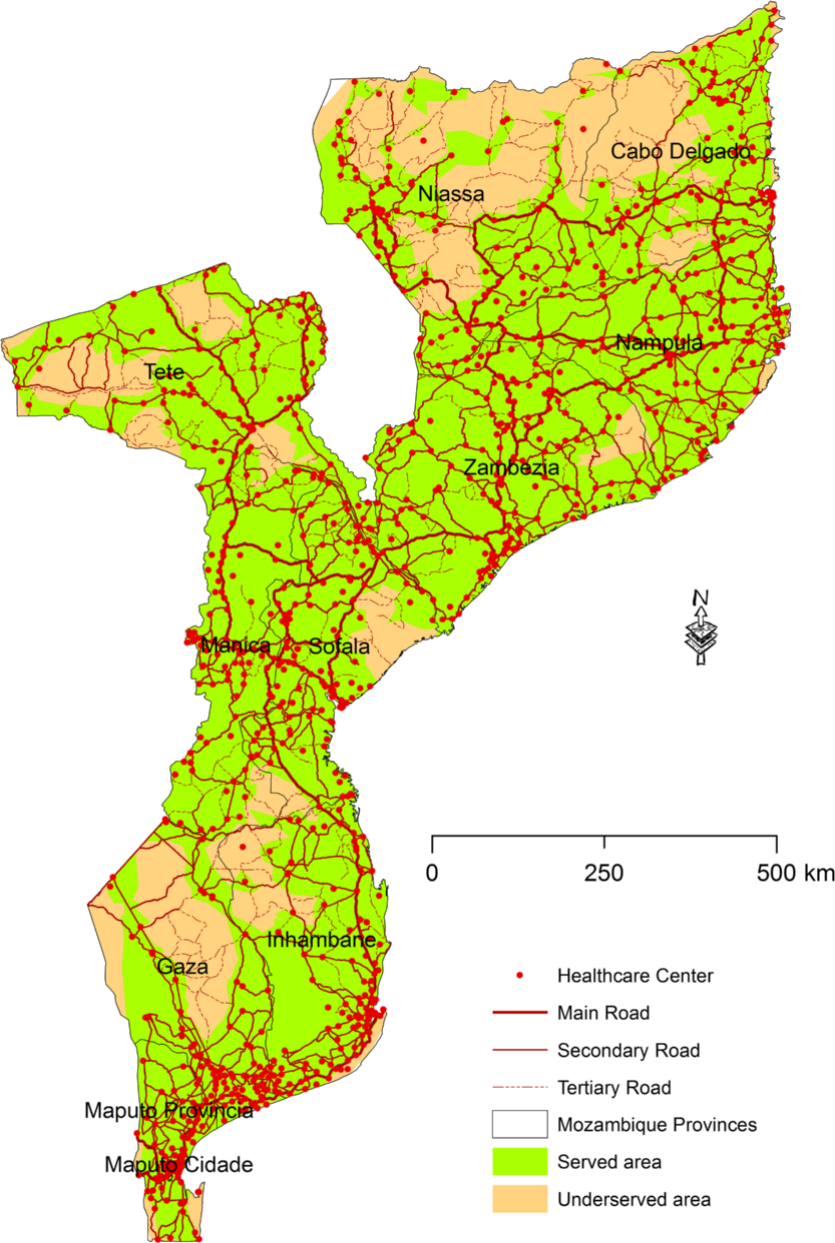
Served and underserved area of Mozambique by Healthcare Centers by driving

The reclassification of the distances to identify the areas served and underserved by HC revealed two classes of distances: served area (0–60 min) and underserved area (more than 60 min) (Fig. 4).

**Fig. 5:**
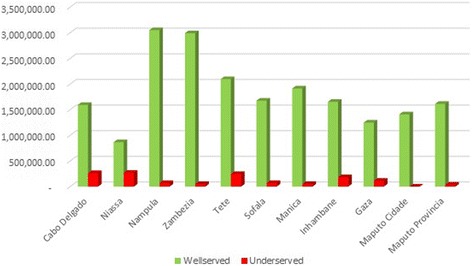
Population Number on the served and underserved areas by HC in the driving scenario

Superposing the areas obtained in the previous map with the projected population data for year 2015 allowed us to obtain the number of population by province: 20,106,550 (93.8 %) people living in the well served area, and 1,345,088 (6.2 %) living in the underserved area. Nampula, Zambezia, Tete, and Manica are the provinces with the highest number of population in the served areas (Fig. 5). Cabo Delgado, Niassa, and Tete are the provinces with the highest number of underserved population, which contrasts with Maputo Cidade, and Province with very low values of people in this condition. Tete is (paradoxically) in both “served” and underserved” areas.

**Fig. 6:**
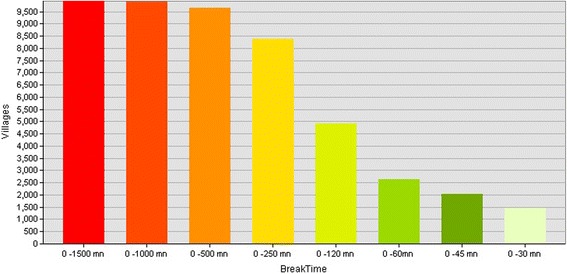
Number of villages per walking time category

**Fig. 7:**
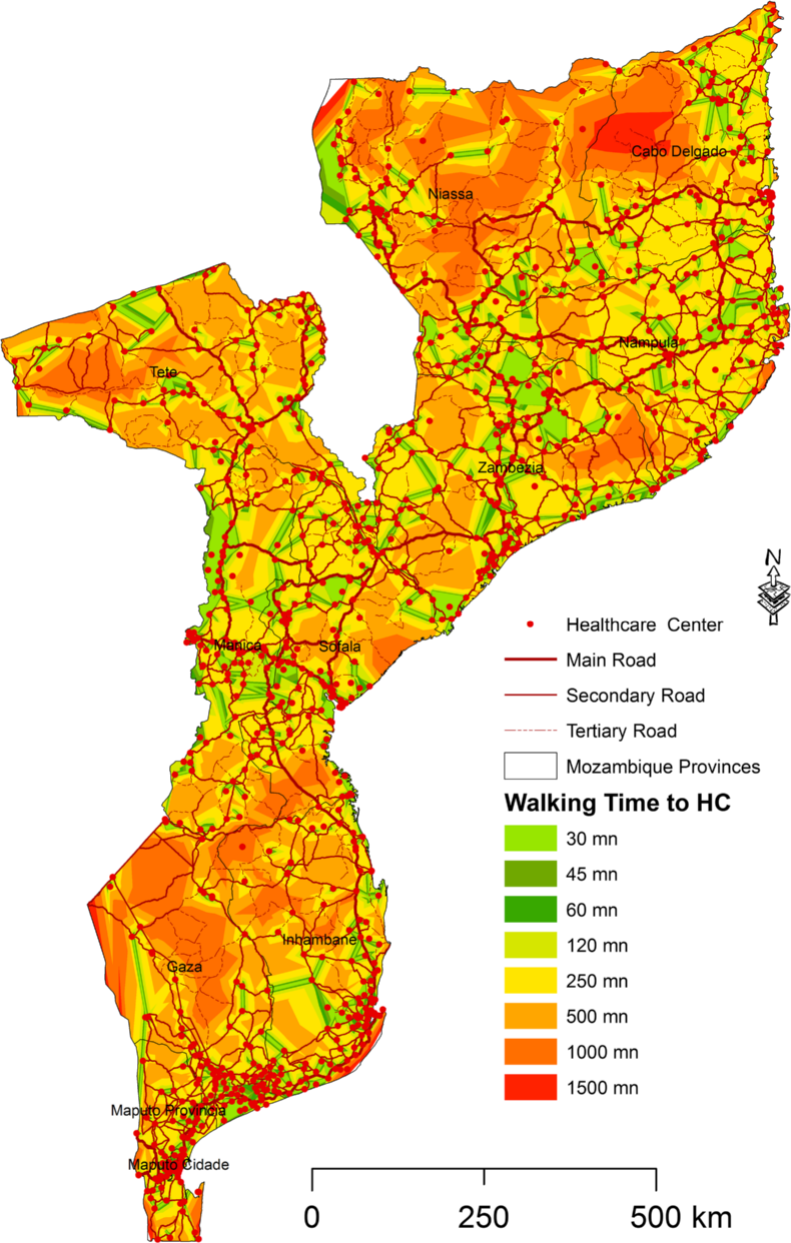
Walking time to Healthcare Centers in different time categories

For the walking scenario, and using the same time breaks as in the previous scenario, we found that there are 1,460 villages located within the distance of 30mn, representing 3 % of the total number of villages (Fig. 6). This number increases slightly to 2,023 within 45mn to the HC, i.e. 4.1 % of the total. Most of the population can reach an HC only if they walk more than 60 min (87.5 %). Fig. 7 shows the SA for walking time in Mozambique.

**Fig. 8:**
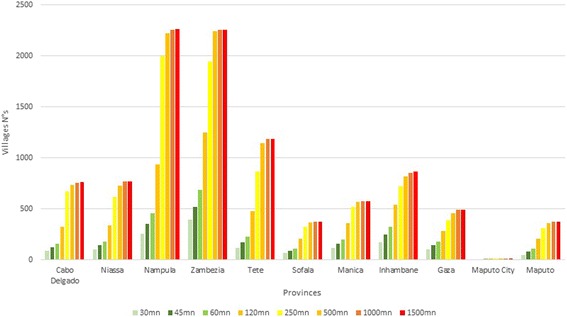
Number of villages per province and walking time categories

An analysis to determine the number of villages per province in each time category was also carried out (Fig. 8). The provinces of Nampula (north), Zambezia and Tete (center), and Inhambane (south) have the highest number of villages outside 60 min from an HC. Maputo, Maputo city, and Sofala are the provinces with the lowest number of villages located outside 60 min from an HC.

**Fig. 9:**
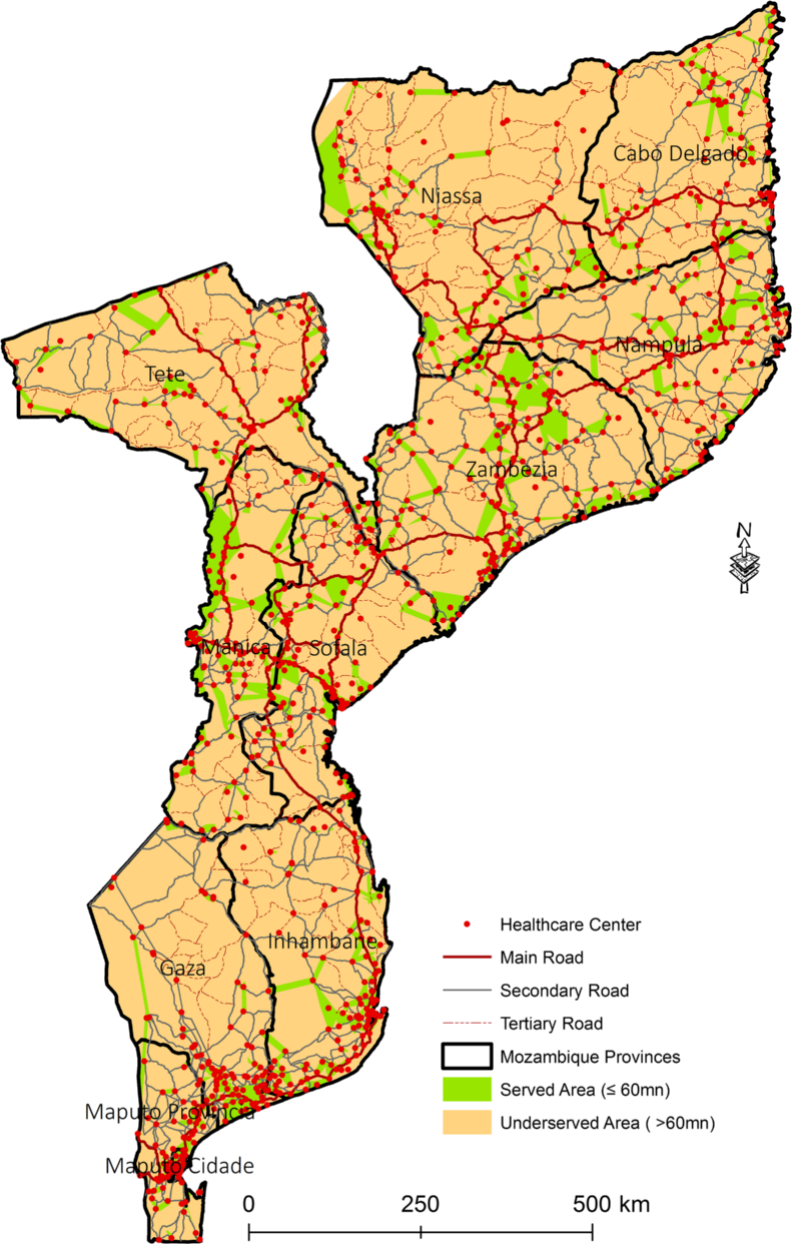
Served and underserved area of Mozambique by Healthcare Centers by walking

The reclassification of the distances to identify served and underserved areas by HC revealed two classes: well served areas (0–60 min) and underserved areas (more than 60 min) (Fig. 9).

**Fig. 10:**
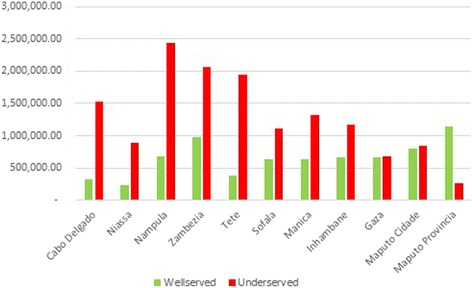
Population in served and underserved areas by Healthcare Centers in the walking scenario

About 7,151,066 (33.3 %) of Mozambicans are living in a served area, while the remaining population, 14,300,572 (66.7 %) are living in an underserved area. Maputo, Zambezia, and Maputo City are the provinces with the highest number of people in the area considered well served regarding the walking time to HC (Fig. 10). Nampula, Zambezia, and Tete are the provinces with the highest number of underserved people, contrary to Maputo, Maputo City, and Gaza with very low values of people in this condition.

## Discussion

This study identifies critical areas in Mozambique where HC may need to be relocated using realistic travel time estimates of driving and walking. In the line of several studies stating that the population should have access to a health facility within one hour of walking, our analysis also uses 60 min as the maximum travelling time [38]. In both scenarios, the areas that can be accessed in more than one hour were classified as underserved area. The findings from this study highlight problems, especially in the walking scenario, in which 90.2 % of Mozambique was considered an underserved area. For the driving scenario, about 66.9 % of Mozambique was considered a served area. Maputo City (100 %), Maputo (90.7 %), and Zambezia (82 %) are the provinces with greatest coverage of HC network. Niassa (62.1 %), Gaza (52.9 %), and Cabo Delgado (48.3 %) are the most underserved provinces. Niassa and Gaza are the two provinces with a negative value for the difference between served and underserved area, i.e., the underserved area is greater than the served area. This can be explained by the reduced number of roads and their poor condition. For the walking scenario, only 9.8 % of Mozambique was considered a served area. Maputo City (69.8 %), Manica (15.8 %), and Zambezia (15.4 %) are the provinces with greatest coverage of HC network. Tete (93.4 %), Cabo Delgado (93 %), and Gaza (92.8 %) provinces are the provinces most underserved. This, as in the driving scenario, can also be related to the reduced number of roads and their poor condition. Only Gaza province has a positive value of the difference between served and underserved area, i.e. the underserved area is smaller than the served area.

Regarding the population distribution (Table 2), we found that the problem of accessibility is mainly in the walking scenario; about 66.7 % of the Mozambican area is located in an underserved area. The accessibility problem is less important than in the scenario of driving (6.27 %). However, there are not many people using their own vehicles or public transportation, especially in the rural areas of the country, where there is a lack of infrastructures and motorized transport services.

**Table 2:** Summary of the population distribution in the two scenarios

	Scenario 1-Driving		Scenario 2-Walking	
	Population		Population	
	N°	%	N°	%
Population Served (≤60 mn)	20,106,550.88	93.73	7,151,066.40	33.3
Underserved Population (>60 mn)	1,345,087.65	6.27	14,300,571.40	66.7
Total	21,451,638.53	100	21,451,637.80	100

The present study has important limitations. First, there is no updated national database of health facilities, although there has been an increase in the number of HC since year 2000. We georeferenced the new HC from the list of recent health facilities (without coordinates) obtained from the Minister of Health of Mozambique. This process was based on the name of the HC and the corresponding name of the villages. Thus, the new HC with names different from the village were not included (there were 245 HC in this situation, representing 18.7 % of the total). We believe both these concerns conservatively biased our estimates of travel times and distances to HC. Second, we are aware that the physical access to HC is only one component of access to healthcare. Factors such as perceived quality of healthcare services, trust in the healthcare providers, quality of and sensitivity in communication by care providers with the public, and ability to pay for the services [39] are potentially determinants to healthcare access that are not addressed in this study. Third, although we used realistic travel time in our analysis, further adjustments may be necessary. For instance, walking speed varies depending on age and the type of individuals involved in the trip (slower for sick adults and adults carrying children compared with adults walking on their own [27, 38]. Therefore, it would be useful to consider these elements for calculating travel times in future studies. In addition, it would be important to incorporate travel cost to identify areas where costs act as obstacles for the health accessibility [40].

Despite these limitations, the present study has several strengths. We estimated travel times and distances using road networks, avoiding straight-line distances. Road travel time estimations produce more accurate results than straight-line distance models because people are inclined to use road networks rather than travel in a straight line [41]. We used geographic locations for each HC as opposed to the approximate locations at district level. We also used population data which is not assigned to the administrative level, avoiding the problems of using aggregated data. Finally, we reported results at national and province levels allowing for the identification of regional disparities.

We have also made some assumptions, including that patients will always travel to the nearest HC. Notwithstanding, they may wish to use more distant care facilities thought to provide better quality services. Another assumption is that travel happens along an optimum path, but due to habits, social factors, environmental and surface conditions, or other costs, some part of the population may prefer to use other routes [42].

## Conclusions

This paper has measured the travel time from any point in Mozambique to its closest HC using two different scenarios and provided new insights about the accessibility to healthcare services in the country. The results of this research show that in terms of geographical accessibility, walking is the most problematic and worrying scenario because the majority of the Mozambican population need 60 min or more to reach an HC.

The findings from this study highlight accessibility problems that are similar to those faced by many African countries [38, 43, 44]. The dissatisfaction caused by distance and long travel time to benefit from healthcare influences the way people respond to the healthcare system in most African countries [45]. People can be frustrated and with negative perceptions of their service providers when they are facing long waiting times to access healthcare services [45]. These results are completely opposite to those of developed countries such as France, where people can access hospital care in less than 45 min, and 75 % in less than 25 min [46].

Our findings may have policy implications for strategies and could be used for advocacy and presentations to donor partners and government, to improve the universal access to the health coverage [1]. In Mozambique, improving the accessibility to health facilities could be achieved in three ways: the first involves the creation of new HC or the reallocation of some HC to maximize the accessibility; the second involves optimizing the public transport network, adapting the offer to the population needs; the third involves the construction of new roads and the rehabilitation of existing roads (the majority of roads are unpaved in rural areas). This integrated view is essential to address the inequalities that arise in the territories, making access to health services more equitable.

## Abbreviations

GIS: Geographic information system
GPW: Gridded population of the world
GRUMP: Global rural–urban mapping project
HC: Healthcare centers
SA: Service area
WHO: World Health Organization
